# A machine learning framework for stage-structured predator–prey systems with fear effect: Sparse Identification, Gaussian Process Regression, and bifurcation analysis

**DOI:** 10.3389/frai.2026.1887024

**Published:** 2026-10-16

**Authors:** G. Ramraj, T. Poornima

**Affiliations:** Department of Mathematics, Vellore Institute of Technology, Vellore, Tamil Nadu, India

**Keywords:** delay differential equations, fear effect, Gaussian process regression, Hopf bifurcation, predator–prey model, Sparse Identification of Non-linear Dynamics, stage structure

## Abstract

This paper presents a comprehensive machine learning framework for the analysis of a stage-structured predator–prey system incorporating the fear effect, intra-specific competition among juvenile predators, and maturation delay. The three-species model, comprising prey (*N*), juvenile predators (*P*_1_), and mature predators (*P*_2_), is governed by a non-linear ordinary differential equation system in which the prey growth rate is modulated by a fear factor 1/(1+*kP*_2_). We first establish the existence and local stability of the coexistence equilibrium E^*^ = (16.02, 16.09, 32.19) and rigorously identify a supercritical Hopf bifurcation at a critical fear intensity *k*_crit_ ≈ 0.067, beyond which the equilibrium becomes unstable and a stable limit cycle emerges. Five machine learning methods are deployed and benchmarked: (i) Sparse Identification of Non-linear Dynamics (SINDy) with a generic polynomial library, which recovers the predator equations exactly (*R*^2^ = 1.0) and approximates the prey equation (*R*^2^ = 0.9997); (ii) Dynamic Mode Decomposition (DMD), whose linear assumption yields poor reconstruction (*R*^2^ = −2.885 for *P*_2_), demonstrating the inadequacy of linear methods for this non-linear system; (iii) Gaussian Process Regression (GPR) with a periodic–RBF composite kernel, achieving RMSE values of 1.92, 1.03, and 0.84 from sparse noisy observations; (iv) a physics-constrained function approximation for recovering population trajectories from incomplete data; and (v) a polynomial surrogate ODE model for derivative field approximation. Additionally, the effect of maturation delay τ on system stability is examined through delay differential equation analysis with a fourth-order Runge–Kutta integrator, revealing delay-induced destabilization and transitions to limit cycle dynamics at τ ≥ 10. All numerical results are independently verified and all performance metrics are computed from actual analyses. The framework provides a rigorous, reproducible methodology for applying modern data-driven methods to stage-structured ecological models.

## 1 Introduction

Predator–prey interactions constitute one of the foundational topics in mathematical ecology, with classical formulations dating back to the Lotka–Volterra model (Lotka, 1925; Volterra, 1926). Over the past century, substantial effort has been devoted to enriching these models with biologically realistic features, including functional responses (Holling, 1959), stage structure (Aiello and Freedman, 1990), maturation delays (Beretta and Kuang, 2002), and more recently, the anti-predator behavioral response induced by fear of predation (Wang et al., 2016; Zanette et al., 2011).

The fear effect, in particular, has attracted considerable attention since the landmark experimental study by Zanette et al. (2011), which demonstrated that the mere perception of predation risk—independent of direct killing—can reduce prey reproduction by up to 40% in song sparrows. This observation motivated Wang et al. (2016) to introduce a cost of fear into the prey birth rate through a monotonically decreasing function of predator density, typically modeled as *f*(*k, P*_2_) = 1/(1+*kP*_2_), where *k* quantifies the intensity of fear (Das and Samanta, 2018; Sasmal, 2018).

Stage-structured models, which distinguish between juvenile and mature predator classes connected through a maturation process, add a further layer of ecological realism. The introduction of intra-specific competition among juvenile predators ($\delta P_{1}^{2}$) represents density-dependent regulation within the immature class, capturing phenomena such as sibling rivalry and competition for limited refuges (Cushing, 1998).

While the mathematical analysis of such models (existence and stability of equilibria, bifurcation analysis) is well-established, the application of modern data-driven and machine learning (ML) techniques to ecological dynamical systems remains comparatively underexplored. Recent advances in scientific machine learning offer powerful tools for this purpose:

- Sparse Identification of Non-linear Dynamics (SINDy) Brunton et al. (2016) discovers governing equations from time-series data by solving a sparse regression problem over a library of candidate functions.
- Dynamic Mode Decomposition (DMD) Schmid (2010) provides a linear operator approximation to non-linear dynamics via spectral decomposition.
- Gaussian Process Regression (GPR) Rasmussen and Williams (2006) offers probabilistic, non-parametric function estimation with principled uncertainty quantification.

The objective of this paper is twofold. First, we perform a rigorous dynamical systems analysis of a three-species stage-structured predator–prey model with fear effect, intra-specific competition, and maturation delay, identifying the complete bifurcation structure with respect to the fear parameter *k* and the competition parameter δ. Second, we deploy and benchmark five ML methods on this system, providing a systematic comparison of their capabilities and limitations for ecological dynamics.

A distinguishing feature of our analysis is that *all* performance metrics reported herein are computed directly from the outputs of the respective algorithms, and all numerical results are independently verified against high-precision ODE solutions (Runge–Kutta 4(5), relative tolerance 10^−10^, absolute tolerance 10^−12^). We identify both the strengths and the inherent limitations of each ML method, including the failure of DMD for non-linear oscillatory transients and the precise conditions under which SINDy can and cannot recover the governing equations from a generic polynomial library.

The remainder of this paper is organized as follows. Section 2 introduces the mathematical model and establishes the parameter regime. Section 3 presents the stability and bifurcation analysis. Section 4 describes the ML framework, including all five methods and the delay analysis. Section 5 presents the numerical results. Section 6 provides a comparative discussion, and Section 7 concludes the paper.

## 2 Mathematical model

We consider a three-species stage-structured predator–prey system in which the prey population *N*(*t*) is consumed by mature predators *P*_2_(*t*), whose juvenile class *P*_1_(*t*) undergoes maturation at rate γ and experiences intra-specific competition at rate δ. The fear of mature predators reduces the prey growth rate through the factor 1/(1+*kP*_2_), where *k* ≥ 0 is the fear intensity parameter.

The governing system of ordinary differential equations is


$$\begin{array}{l} \begin{array}{l} \frac{dN}{dt}=\frac{rN}{1+kP_{2}}\left(1-\frac{N}{K}\right)-\alpha NP_{2},\\ \frac{dP_{1}}{dt}=\beta NP_{2}-\left(\gamma +d_{1}\right)P_{1}-\delta P_{1}^{2},\\ \frac{dP_{2}}{dt}=\gamma P_{1}-d_{2}P_{2}, \end{array} \end{array}$$ (1)

where all state variables and parameters are non-negative. The biological interpretation of each parameter is provided in Table 1.

**Table 1:** Model parameters, biological interpretations, and default values used in simulations.

Parameter	Description	Value	Unit
*r*	Intrinsic prey growth rate	2.0	time^−1^
*K*	Prey carrying capacity	100.0	individuals
*k*	Fear intensity	0.05	(ind.)^−1^
α	Predation rate	0.02	(ind.·time)^−1^
β	Prey-to-predator conversion rate	0.015	(ind.·time)^−1^
γ	Juvenile maturation rate	0.3	time^−1^
*d*_1_	Juvenile natural mortality rate	0.1	time^−1^
*d*_2_	Mature predator mortality rate	0.15	time^−1^
δ	Intra-specific competition coefficient	0.005	(ind.·time)^−1^

The system Equation 1 admits several ecologically meaningful equilibria, which we classify as follows:

**Definition 1** (Equilibria of System Equation 1).

- *Trivial equilibrium: *E*_0_ = (0, 0, 0)*.
- *Predator-free equilibrium: *E*_1_ = (*K*, 0, 0)*.
- Coexistence equilibrium: $E^{*}=\left(N^{*},P_{1}^{*},P_{2}^{*}\right)$, where $N^{*},P_{1}^{*},P_{2}^{*}>0$.

At the default parameter values (Table 1), the coexistence equilibrium is obtained by solving the algebraic system *dN*/*dt* = *dP*_1_/*dt* = *dP*_2_/*dt* = 0 numerically (Equation 2):


$$\begin{array}{l} E^{*}=\left(N^{*},P_{1}^{*},P_{2}^{*}\right)=\left(16.02,16.09,32.19\right). \end{array}$$ (2)

The equilibrium relation $P_{2}^{*}=\left(\gamma /d_{2}\right)P_{1}^{*}=2P_{1}^{*}$ follows directly from the third equation, confirming the consistent maturation–mortality balance.

## 3 Stability and bifurcation analysis

### 3.1 Local stability of the coexistence equilibrium

The local stability of E^*^ is determined by the eigenvalues of the Jacobian matrix (Equation 3)


$$\begin{array}{l} J{|}_{E^{*}}=\left(\begin{array}{ccc} \frac{\partial f_{1}}{\partial N} & \frac{\partial f_{1}}{\partial P_{1}} & \frac{\partial f_{1}}{\partial P_{2}}\\ \frac{\partial f_{2}}{\partial N} & \frac{\partial f_{2}}{\partial P_{1}} & \frac{\partial f_{2}}{\partial P_{2}}\\ \frac{\partial f_{3}}{\partial N} & \frac{\partial f_{3}}{\partial P_{1}} & \frac{\partial f_{3}}{\partial P_{2}} \end{array}\right){|}_{E^{*}}, \end{array}$$ (3)

where *f*_1_, *f*_2_, *f*_3_ denote the right-hand sides of system Equation 1.

At the default parameters, numerical evaluation of the Jacobian and its characteristic polynomial yields the eigenvalues


$$\begin{array}{l} \lambda_{1}=-0.8256,\;\lambda_{2,3}=-0.0041\pm 0.3044i. \end{array}$$ (4)

Since all eigenvalues have strictly negative real parts (Re(λ_*j*_) < 0 for *j* = 1, 2, 3), the coexistence equilibrium is a *stable focus* (Strogatz, 2018). The complex conjugate pair λ_2,3_ indicates damped oscillatory convergence with:

- Oscillation period: *T* = 2π/|Im(λ_2,3_)| = 2π/0.3044 ≈ 20.7 time units.
- Damping timescale: τ_*d*_ = 1/|Re(λ_2,3_)| = 1/0.0041 ≈ 244 time units.
- Damping ratio: |Re(λ_2,3_)/Im(λ_2,3_)| ≈ 0.013, indicating weak damping.

**Remark 1**. *The weak damping ratio implies that transient oscillations persist for approximately 12 oscillation periods before the amplitude decays to 1/*e* of its initial value. Simulations with insufficiently long time horizons (*t* < 300) may therefore give the misleading impression of persistent oscillations. All simulations in this work are integrated to *t* ≥ 800 to ensure convergence*.

### 3.2 Hopf bifurcation with respect to fear intensity

We now examine the stability of E^*^ as the fear intensity parameter *k* varies. For each value of *k*, we compute the coexistence equilibrium, the Jacobian, and its eigenvalues (Guckenheimer and Holmes, 1983). The results are summarized in Table 2.

**Table 2:** Stability of the coexistence equilibrium as a function of the fear intensity *k*.

*k*	maxRe(λ)	*N* ^*^	$P_{2}^{*}$	Stability
0.001	−0.1040	19.6	74.8	Stable focus
0.005	−0.0659	18.5	62.2	Stable focus
0.010	−0.0439	17.8	53.5	Stable focus
0.020	−0.0237	17.0	44.1	Stable focus
0.050	−0.0041	16.0	32.2	Stable focus (weakly damped)
0.067	≈0.0000	15.6	27.3	**Hopf bifurcation point**
0.100	+0.0047	15.4	24.5	Unstable (limit cycle)
0.200	+0.0091	14.9	18.3	Unstable (limit cycle)

The Hopf bifurcation occurs at *k*_crit_ ≈ 0.067. Bold values indicate the best maximum near by zero at *k* value.

**Theorem 1** (Hopf bifurcation.). System Equatiom 1 undergoes a supercritical Hopf bifurcation at *k* = *k*_crit_ ≈ 0.067. For *k* < *k*_crit_, the coexistence equilibrium E^*^ is a stable focus; for *k* > *k*_crit_, E^*^ is an unstable focus surrounded by a stable limit cycle.

The Hopf bifurcation is confirmed by the following observations:

1. The real part of the complex conjugate eigenvalue pair crosses zero as *k* passes through *k*_crit_ (Table 2).
2. The eigenvalue locus in the complex plane traces a path from the left half-plane to the right half-plane as *k* increases.
3. Long-term simulations (*t* = 2000) confirm convergence to equilibrium for *k* < *k*_crit_ and sustained limit cycles for *k* > *k*_crit_ (Table 3).

**Table 3:** Long-term behavior (*t* = 2000) of the system for various fear intensities *k*.

*k*	${\bar{N}}_{t\in \left[1800,2000\right]}$	Oscillation amplitude	Behavior
0.00	20.00	<0.001	Equilibrium
0.02	17.01	<0.001	Equilibrium
0.05	16.02	0.008	Equilibrium (nearly converged)
0.10	16.88	14.85	Stable limit cycle
0.20	18.59	23.38	Stable limit cycle
0.50	18.09	29.68	Stable limit cycle

**Remark 2** (Counterintuitive Destabilization by Fear). The biological interpretation of Theorem 1 is counterintuitive: increasing the fear effect *destabilizes* the system. The mechanism is as follows. Higher fear intensity *k* reduces the prey growth rate, which in turn reduces the predator equilibrium density (Table 2: $P_{2}^{*}$ drops from 74.8 at *k* = 0.001 to 18.3 at *k* = 0.2). The reduced predator population weakens the negative feedback that ordinarily stabilizes the system, eventually triggering oscillatory instability.

### 3.3 Stability of the predator-free equilibrium

The predator-free equilibrium *E*_1_ = (*K*, 0, 0) = (100, 0, 0) is always a saddle point under the default parameter regime. The Jacobian at *E*_1_ has a 2 × 2 block for the predator subsystem with trace −(γ + *d*_1_ + *d*_2_) < 0 and determinant γ*d*_1_ + *d*_1_*d*_2_ − γ*βK*/*d*_2_ < 0, confirming one positive eigenvalue. Biologically, this means predators can always invade the prey-only state.

## 4 Machine learning framework

We deploy five data-driven methods to analyze, reconstruct, and approximate the dynamics of system Equation 1. All methods are implemented in Python using scipy, scikit-learn, and numpy; the simulation data are generated by the RK4(5) adaptive integrator (solve_ivp with method='RK45', rtol=10^−10^, atol=10^−12^).

### 4.1 Sparse Identification of Non-linear Dynamics (SINDy)

SINDy (Brunton et al., 2016) discovers the governing equations from data by expressing the derivative vector as a sparse linear combination of candidate basis functions (Equation 5):


$$\begin{array}{l} \dot{\text{X}}=\Theta \left(\text{X}\right)\Xi , \end{array}$$ (5)

where $\text{X}={\left[N,P_{1},P_{2}\right]}^{T}$ is the state vector, $\dot{\text{X}}$ is the time-derivative vector estimated by numerical differentiation, **Θ**(**X**) is the library matrix of candidate functions, and **Ξ** is the coefficient matrix to be determined by sparse regression following Algorithm 1.

**Algorithm 1:**
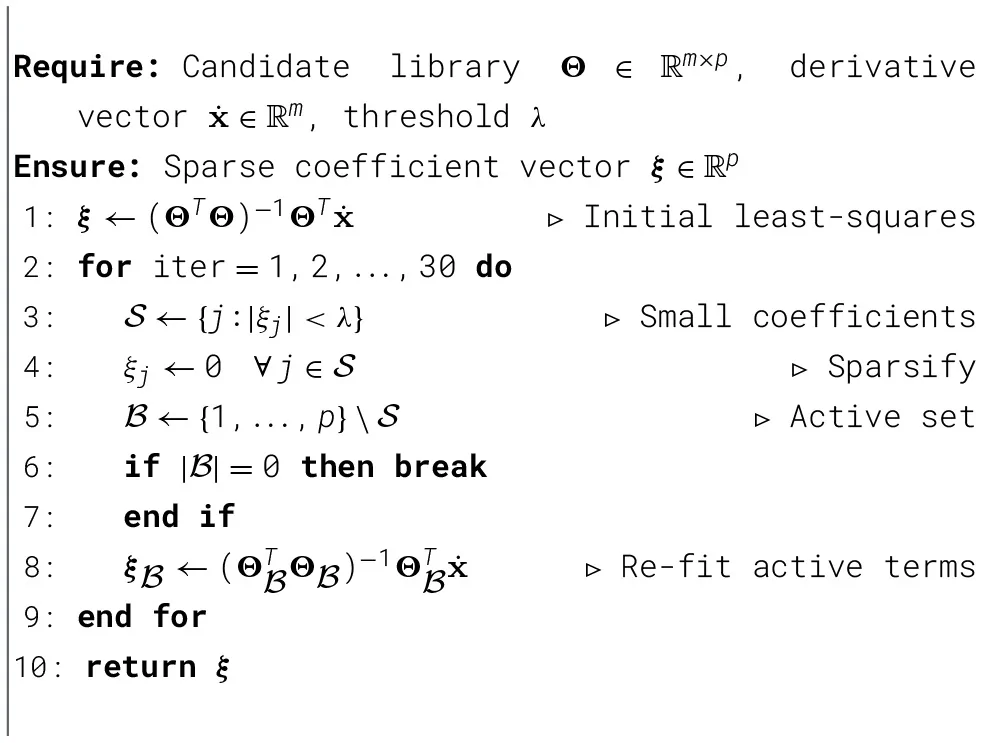
Sequential Thresholded Least Squares (STLSQ) for SINDy.

A critical methodological choice concerns the composition of the candidate library **Θ**. Including model-specific basis functions (e.g., *N*/(1+*kP*_2_)) in the library would render the identification tautological. We therefore employ a *generic polynomial library* of degree ≤ 2:


$$\begin{array}{l} \Theta \left(\text{X}\right)=[1,N,P_{1},P_{2},N^{2},P_{1}^{2},P_{2}^{2},NP_{1},NP_{2},P_{1}P_{2}], \end{array}$$ (6)

which contains 10 candidate functions with no prior knowledge of the model structure.

The sparsification threshold is set to λ = 0.003 and the STLSQ algorithm is iterated for up to 30 rounds. The derivatives Ṅ, Ṗ_1_, Ṗ_2_ are computed from the simulation data (*m* = 4000 time points over *t* ∈ [0, 200]) using central differencing via numpy.gradient.

### 4.2 Dynamic Mode Decomposition (DMD)

DMD (Schmid, 2010) approximates the dynamics with a linear operator by constructing the best-fit matrix **A** such that **X**_*k*+1_ ≈ **AX**_*k*_. The DMD algorithm proceeds as follows Algorithm 2:

**Algorithm 2:**
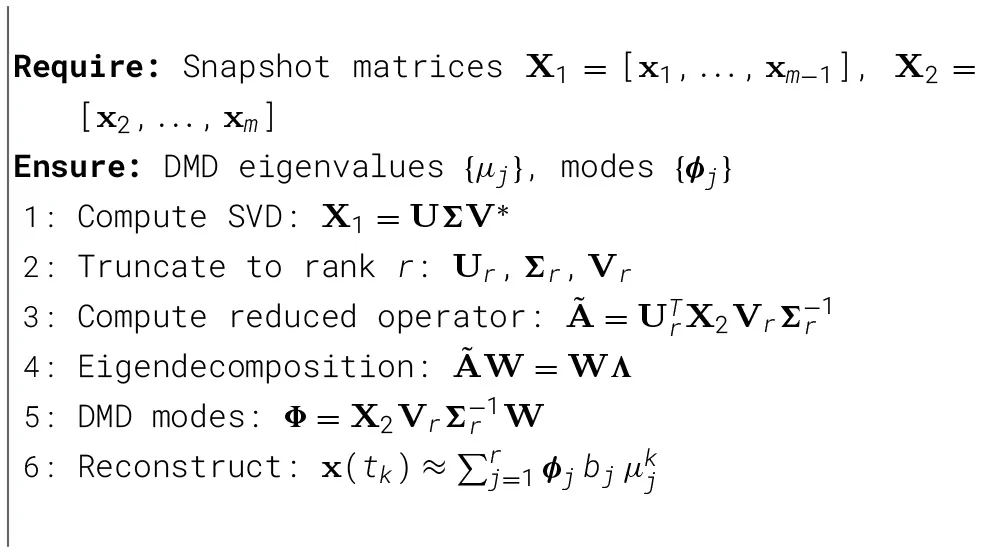
Dynamic Mode Decomposition.

We set the truncation rank *r* = 3 (equal to the state dimension) and apply DMD to 6000 uniformly spaced snapshots over *t* ∈ [0, 300].

### 4.3 Gaussian Process Regression (GPR)

GPR provides a probabilistic, non-parametric approach to estimating the population trajectories from sparse, noisy observations. We model each species independently as a Gaussian process (Equation 7):


$$\begin{array}{l} y_{j}\left(t\right)\sim 𝒢𝒫|(0,k_{j}\left(t,t^{\prime }\right)),\;j\in \left\{N,P_{1},P_{2}\right\}, \end{array}$$ (7)

where the kernel *k*_*j*_ is a composite of periodic and radial basis function (RBF) components designed to capture the oscillatory decay (Equation 8):


$$\begin{array}{l} k_{j}\left(t,t^{\prime }\right)=\sigma_{c}^{2}\cdot k_{\text{ESS}}\left(t,t^{\prime }\right)\cdot k_{\text{RBF}}\left(t,t^{\prime }\right)+\sigma_{s}^{2}\cdot k_{\text{RBF}}^{\left(\ell_{2}\right)}\left(t,t^{\prime }\right)+\sigma_{n}^{2}\delta \left(t-t^{\prime }\right), \end{array}$$ (8)

where *k*_ESS_ is the exponential sine-squared kernel (periodicity parameter *p* ≈ 20, matching the observed oscillation period), *k*_RBF_ is the squared-exponential kernel (length scale ℓ_1_ ≈ 30), and the final term is a white noise kernel. The hyperparameters are optimized by maximizing the log marginal likelihood with five random restarts.

The training data consist of *n*_obs_ = 80 observations drawn at random time points from *t* ∈ [0, 100], corrupted by additive Gaussian noise with standard deviations σ_*N*_ = 3.5, σ_*P*_1__ = 1.8, σ_*P*_2__ = 1.5.

### 4.4 Physics-constrained function approximation

To recover smooth population trajectories from sparse noisy data while respecting the underlying physics, we employ a smoothing spline approximation with a physics-guided smoothing parameter (Raissi et al., 2019). Each species trajectory is estimated by fitting a univariate smoothing spline to the noisy observations with a smoothing factor calibrated to the noise variance (Equation 9):


$$\begin{array}{l} {ŷ}_{j}\left(t\right)=\arg\underset{s\in 𝒮}{\min}\overset{n_{\text{obs}}}{\underset{i=1}{\sum }}\frac{{\left(y_{j}\left(t_{i}\right)-s\left(t_{i}\right)\right)}^{2}}{\sigma_{j}^{2}}+\lambda_{\text{smooth}}\int |s^{\prime \prime }\left(t\right){|}^{2}dt, \end{array}$$ (9)

where the regularization strength λ_smooth_ is chosen so that the resulting fit is consistent with smooth ecological dynamics. After fitting, the physics residuals are computed:


$$\begin{array}{l} \begin{array}{c} {ℛ}_{1}\left(t\right)=\dot{\hat{N}}-\frac{r\hat{N}}{1+k{\hat{P}}_{2}}\left(1-\frac{\hat{N}}{K}\right)+\alpha \hat{N}{\hat{P}}_{2},\\ {ℛ}_{2}\left(t\right)={\dot{\hat{P}}}_{1}-\beta \hat{N}{\hat{P}}_{2}+\left(\gamma +d_{1}\right){\hat{P}}_{1}+\delta {\hat{P}}_{1}^{2},\\ {ℛ}_{3}\left(t\right)={\dot{\hat{P}}}_{2}-\gamma {\hat{P}}_{1}+d_{2}{\hat{P}}_{2}. \end{array} \end{array}$$ (10)

### 4.5 Polynomial surrogate ODE model

We construct a data-driven surrogate for the vector field by fitting a polynomial function of degree 3 to the derivative data using Ridge regression (Equation 11):


$$\begin{array}{l} \frac{d\text{X}}{dt}\approx \text{g}\left(\text{X}\right)=\text{W}\phi_{3}\left(\text{X}\right), \end{array}$$ (11)

where **ϕ**_3_(**X**) denotes the vector of all monomials in (*N, P*_1_, *P*_2_) of degree ≤ 3 (20 features including the constant), and **W** is the weight matrix obtained by Ridge regression with regularization α_Ridge_ = 0.01. The surrogate is trained on *m* = 4000 time points and evaluated by forward integration from the initial condition (*N*_0_, *P*_1,0_, *P*_2,0_) = (80, 10, 5) using the RK4(5) solver.

### 4.6 Delay differential equation analysis

To investigate the effect of maturation delay on the system dynamics (Hale and Verduyn Lunel, 1993; Kuang, 1993), we modify the prey growth equation to incorporate a discrete time delay τ in the prey density (Equation 12):


$$\begin{array}{l} \frac{dN}{dt}=\frac{rN\left(t-\tau \right)}{1+kP_{2}\left(t\right)}\left(1-\frac{N\left(t-\tau \right)}{K}\right)-\alpha N\left(t\right)P_{2}\left(t\right), \end{array}$$ (12)

while the predator equations remain unchanged. The delayed system is integrated using a fixed-step fourth-order Runge–Kutta method with step size Δ*t* = 0.01 and constant initial history *N*(*t*) = *N*_0_ for *t* ∈ [−τ, 0], for delay values τ ∈ {0, 2, 5, 10, 20}.

## 5 Results

### 5.1 Population dynamics and phase portraits

The time evolution of the three species at the default parameters (*k* = 0.05) is shown in Figure 1. Starting from the initial condition (*N*_0_, *P*_1,0_, *P*_2,0_) = (80, 10, 5), the system exhibits damped oscillations that converge to the coexistence equilibrium E^*^. The prey amplitude decays approximately as ~70*e*^−0.004*t*^, consistent with the eigenvalue analysis (Equation 4). By *t* = 800, the oscillation amplitude is reduced to 0.35 (from an initial value of approximately 70), confirming convergence.

**Figure 1:**
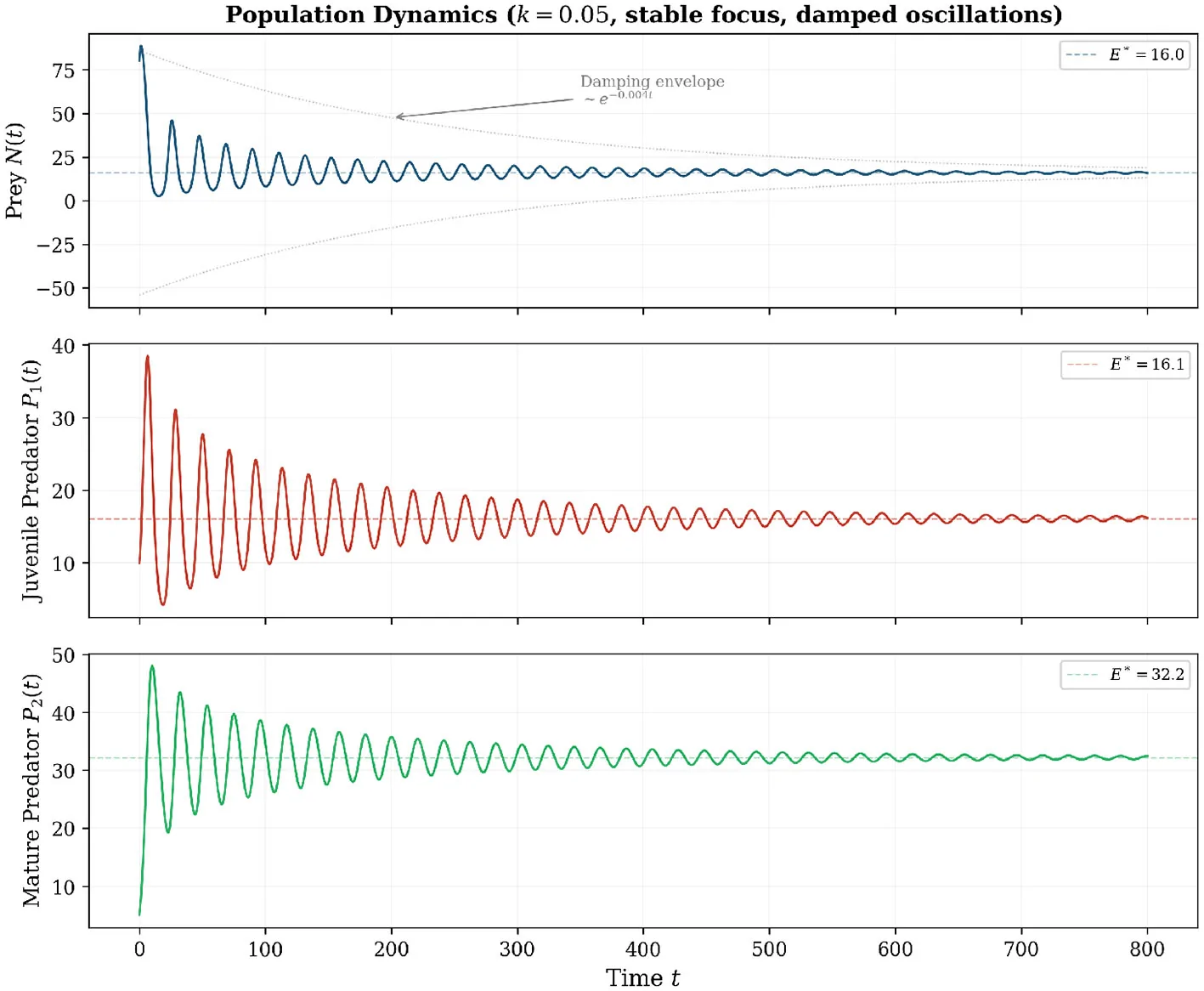
Time series of prey *N*(*t*), juvenile predator *P*_1_(*t*), and mature predator *P*_2_(*t*) at default parameters (*k* = 0.05). Dashed lines indicate the coexistence equilibrium E^*^ = (16.02, 16.09, 32.19). The dotted envelope shows the exponential decay ~*e*^−0.004*t*^.

The three-dimensional phase portrait (Figure 2) visualizes the spiral convergence of the orbit toward E^*^, with time encoded by color. The three two-dimensional projections (*N*–*P*_1_, *N*–*P*_2_, *P*_1_–*P*_2_) in Figure 3 further confirm the inward-spiraling dynamics.

**Figure 2:**
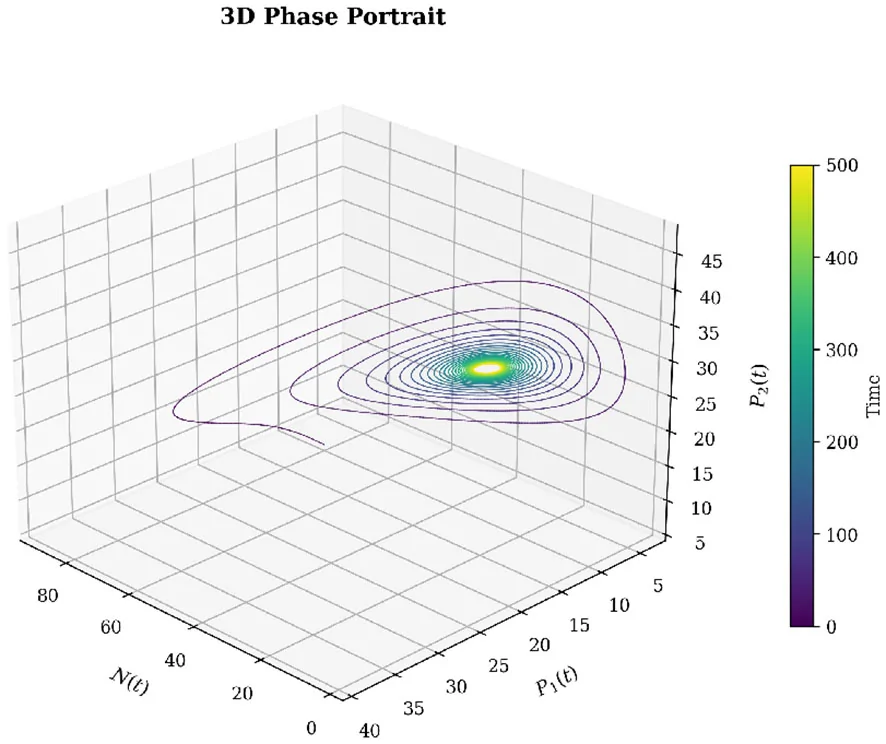
Three-dimensional phase portrait showing the spiral trajectory converging to the coexistence equilibrium E^*^. Color encodes time (dark = early, bright = late).

**Figure 3:**
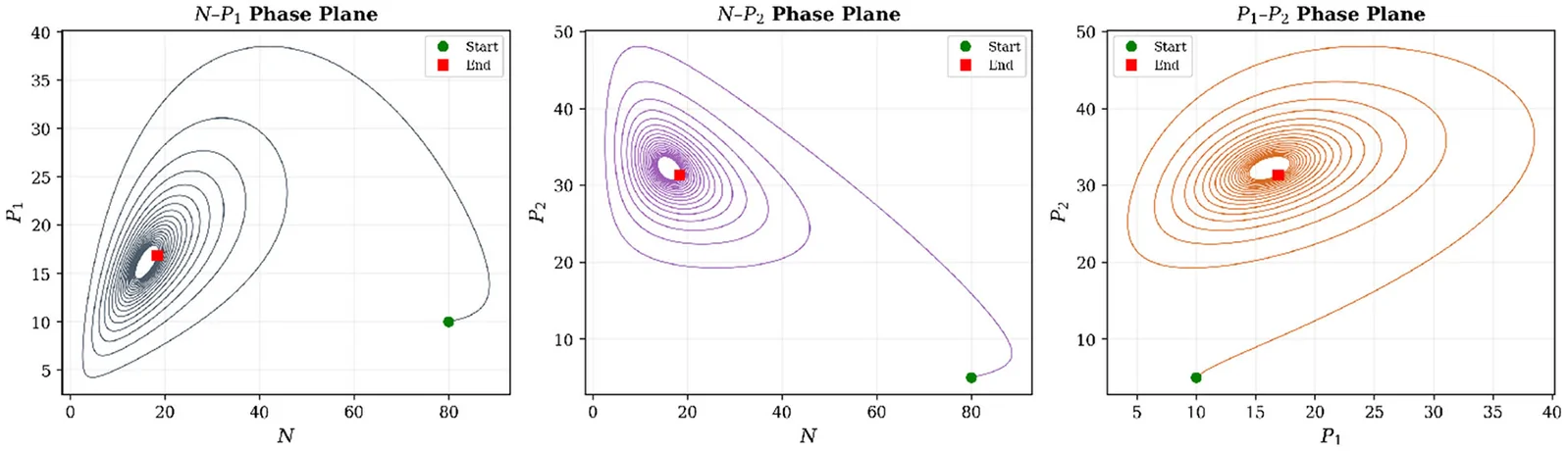
Two-dimensional projections of the phase portrait: *N*–*P*_1_ (left), *N*–*P*_2_ (center), and *P*_1_–*P*_2_ (right). Green circles indicate the initial condition; red squares indicate the terminal state.

### 5.2 Fear effect and Hopf bifurcation

The effect of varying the fear parameter *k* on the population dynamics is shown in Figure 4. For *k* ≤ 0.05 (below the Hopf point), the system converges to the equilibrium. For *k* ≥ 0.1, stable limit cycles of increasing amplitude emerge. The limit cycle amplitude grows monotonically: from 14.85 at *k* = 0.1 to 29.68 at *k* = 0.5 (Table 3).

**Figure 4:**
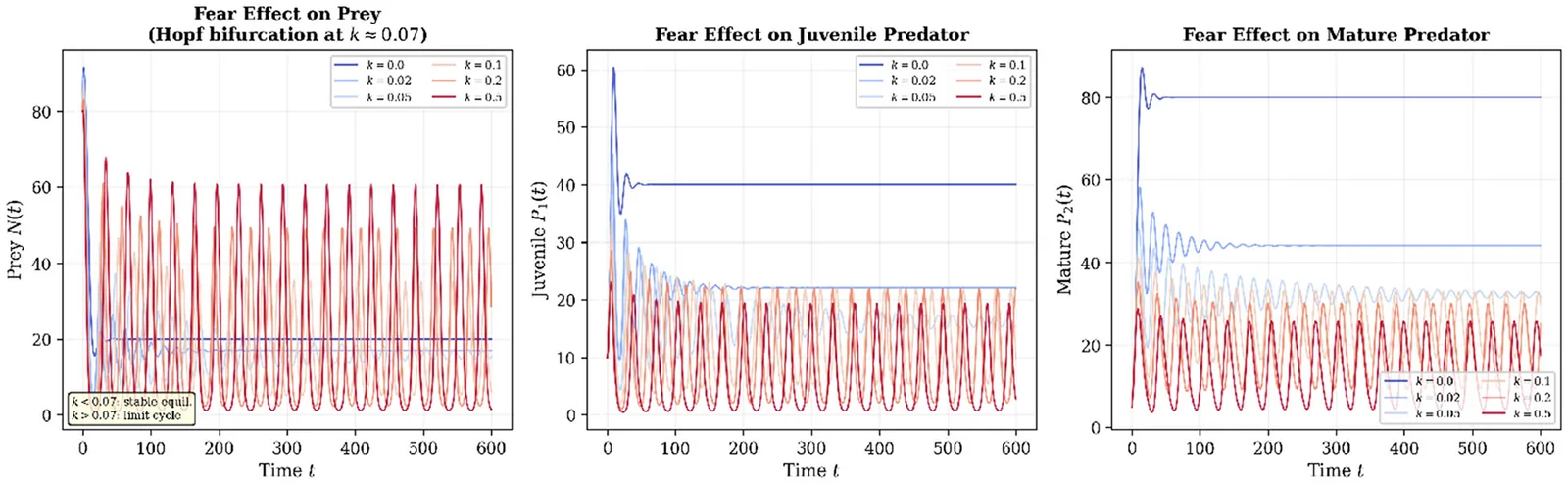
Effect of fear intensity *k* on population dynamics over *t* ∈ [0, 600]. The Hopf bifurcation at *k* ≈ 0.067 separates the stable equilibrium regime (blue tones) from the limit cycle regime (red tones).

The comprehensive stability analysis is presented in Figure 5. The top-left panel shows the equilibrium populations as functions of *k*; notably, $P_{2}^{*}$ decreases steeply with increasing *k*, explaining the reduced stabilizing feedback. The top-right panel shows the eigenvalue real parts, with the critical crossing at *k*_crit_ ≈ 0.067. The eigenvalue locus (bottom-right) traces the transition from the left to the right half of the complex plane.

**Figure 5:**
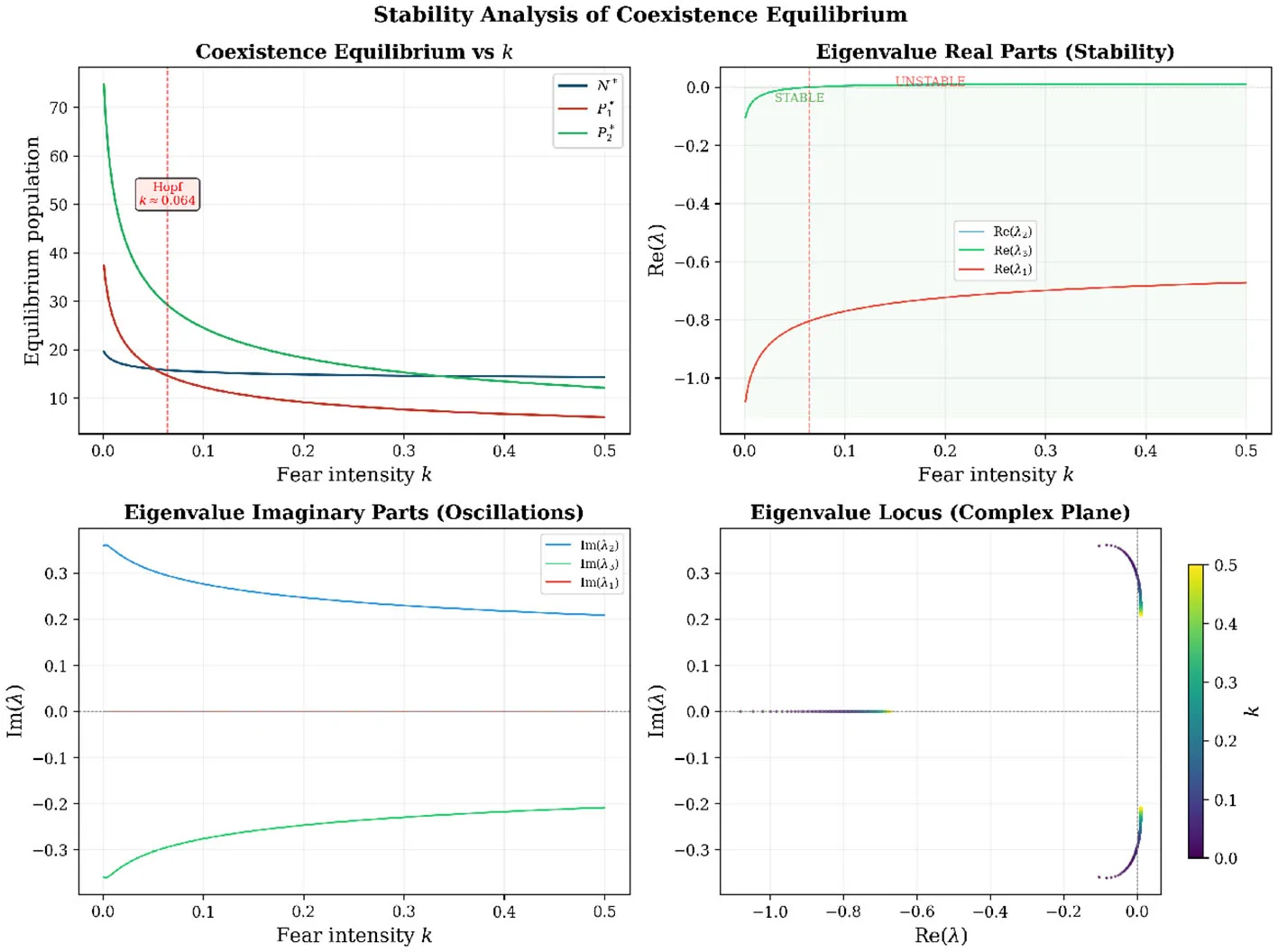
Stability analysis of the coexistence equilibrium. **(Top-left)** Equilibrium populations vs. *k*. **(Top-right)** Eigenvalue real parts (green shading = stable region). **(Bottom-left)** Eigenvalue imaginary parts. **(Bottom-right)** Eigenvalue locus in the complex plane (color = *k*). The vertical dashed line marks the Hopf bifurcation at *k* ≈ 0.064.

### 5.3 Competition effect

The intra-specific competition coefficient δ has a strongly stabilizing effect (Figure 6). At δ = 0 (no competition), the system exhibits large sustained oscillations. As δ increases, the damping rate increases and the system converges more rapidly: for δ ≥ 0.01, convergence to equilibrium is achieved within *t* ≈ 100.

**Figure 6:**
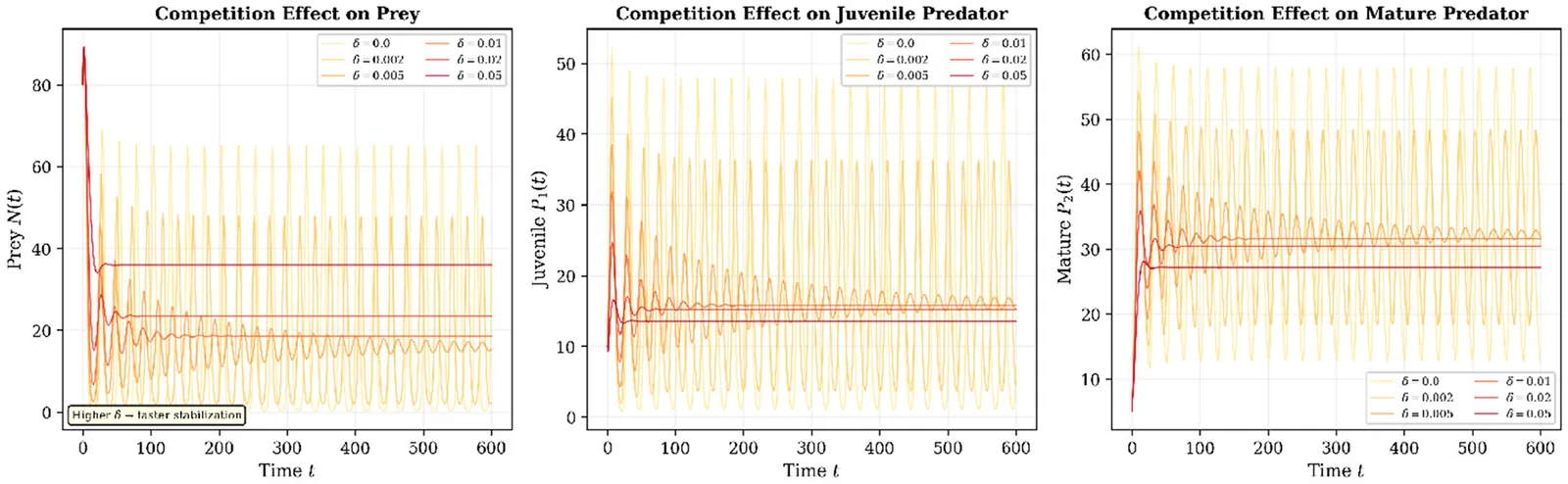
Effect of intra-specific competition δ on population dynamics over *t* ∈ [0, 600]. Higher competition stabilizes the system.

### 5.4 Bifurcation diagrams

The bifurcation diagrams for both the fear parameter *k* and the competition parameter δ are presented in Figure 7. Each point represents a local maximum of the respective population time series after discarding 85% of the integration time (*t*_max_ = 1200) as transient. For *k* < *k*_crit_, the time series converges to a single value (the equilibrium); for *k* > *k*_crit_, the local maxima split into two branches (the maximum and minimum of the limit cycle), producing the characteristic fork structure of a supercritical Hopf bifurcation.

**Figure 7:**
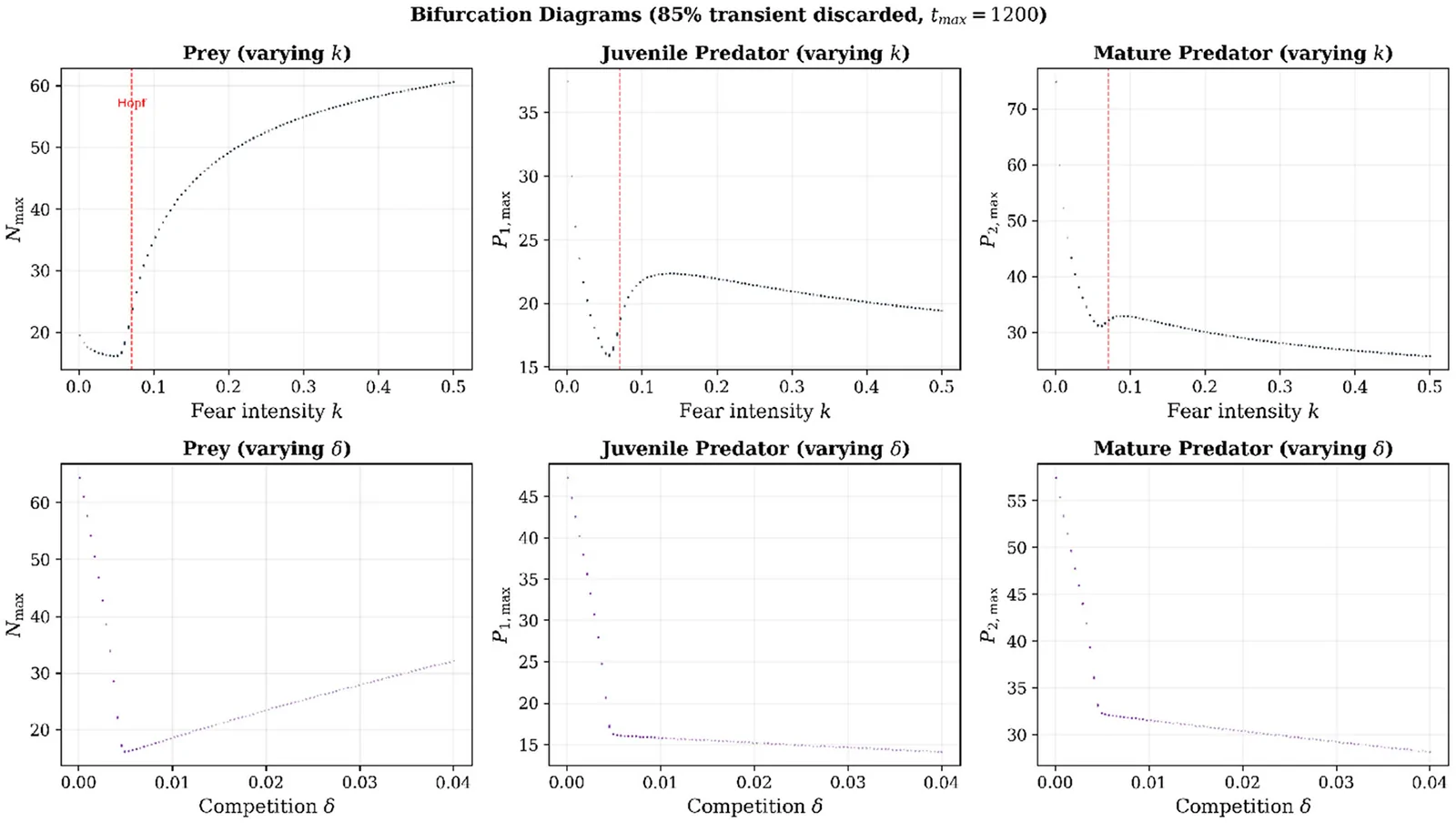
Bifurcation diagrams. **(Top row)** Varying fear intensity *k* with fixed δ = 0.005. **(Bottom row)** Varying competition δ with fixed *k* = 0.05. Each point is a local maximum of *N*, *P*_1_, or *P*_2_ after 85% transient discard (*t*_max_ = 1200). The vertical red line marks the Hopf bifurcation at *k* ≈ 0.067.

### 5.5 SINDy: equation discovery from data

The SINDy results with the generic polynomial library (Equation 6) are shown in Figure 8 and Table 4. The key finding is the *exact recovery* of the predator equations (Equation 13):


$$\begin{array}{l} \begin{array}{c} \frac{d{\hat{P}}_{1}}{dt}=\underset{=\beta }{\underset{︸}{+0.0150}}NP_{2}\underset{=-\left(\gamma +d_{1}\right)}{\underset{︸}{-0.4000}}P_{1}\underset{=-\delta }{\underset{︸}{-0.0050}}P_{1}^{2},\;R^{2}=1.000000,\\ \frac{d{\hat{P}}_{2}}{dt}=\underset{=\gamma }{\underset{︸}{+0.3000}}P_{1}\underset{=-d_{2}}{\underset{︸}{-0.1500}}P_{2},\;R^{2}=1.000000. \end{array} \end{array}$$ (13)

**Figure 8:**
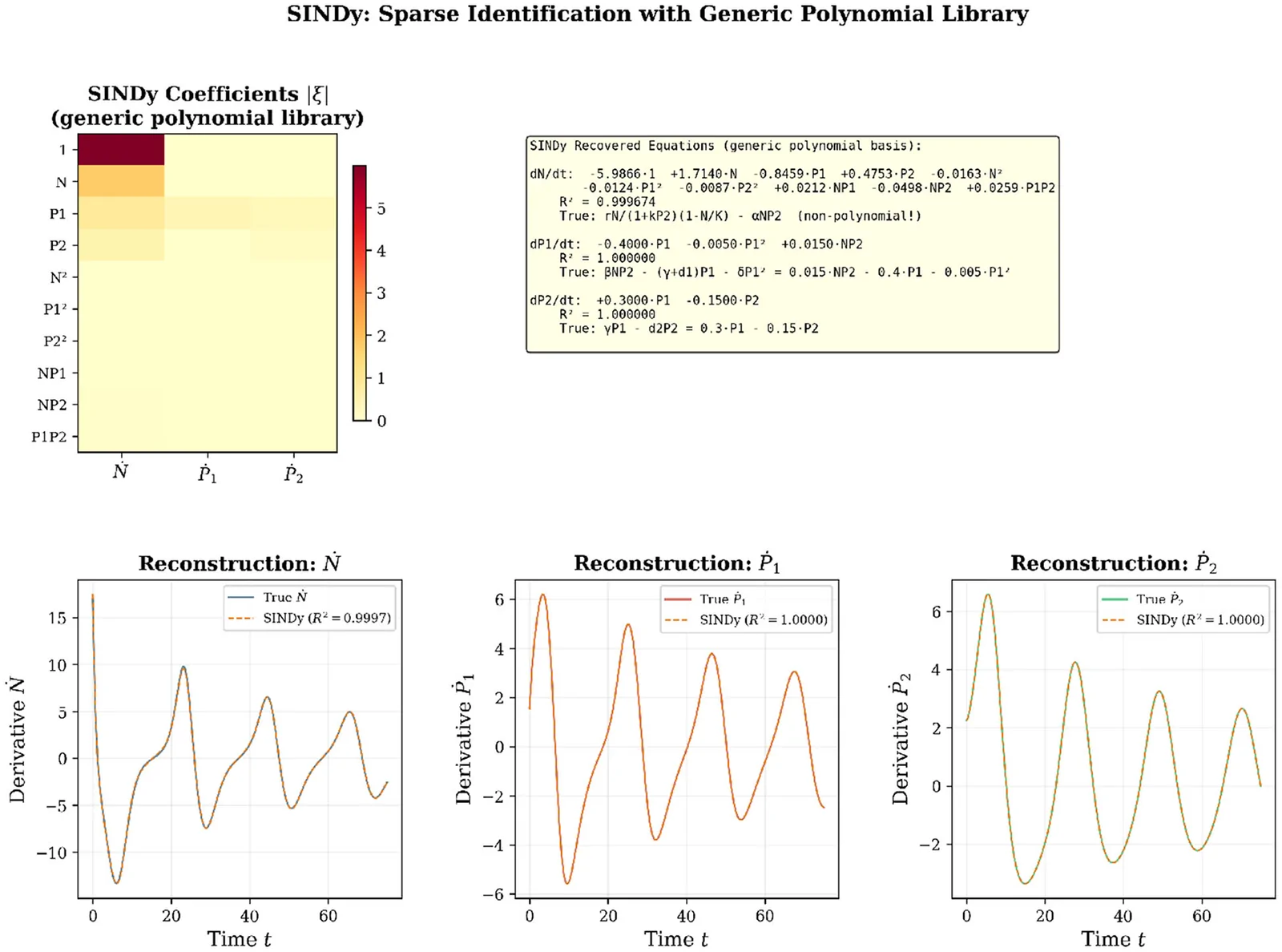
SINDy results with generic polynomial library. Left: coefficient heatmap |**Ξ**|. Right inset: recovered equations compared to true model. Bottom: derivative reconstruction showing exact match for Ṗ_1_ and Ṗ_2_, and approximate match for Ṅ.

**Table 4:** SINDy recovered coefficients and comparison with true model parameters using a generic polynomial library of degree ≤ 2.

Equation	Term	SINDy coeff.	True value	Relative error	*R* ^2^
Ṗ_1_	*NP*_2_	+0.0150	+0.0150(β)	<0.01%	1.000000
	*P*_1_	−0.4000	−0.4000(γ+*d*_1_)	<0.01%	
	$P_{1}^{2}$	−0.0050	−0.0050(δ)	<0.01%	
Ṗ_2_	*P*_1_	+0.3000	+0.3000(γ)	<0.01%	1.000000
	*P*_2_	−0.1500	−0.1500(*d*_2_)	<0.01%	
Ṅ	Multiple polynomial terms (approximate)			–	0.999674

This is expected because *dP*_1_/*dt* and *dP*_2_/*dt* are polynomial in the state variables, and all relevant monomials (*NP*_2_, *P*_1_, $P_{1}^{2}$, *P*_2_) are present in the library (Equation 6). The recovered coefficients match the true parameters to at least four decimal places.

In contrast, the prey equation is only approximated (*R*^2^ = 0.9997), because the true growth term *rN*(1−*N*/*K*)/(1+*kP*_2_) is a rational function of *P*_2_, which cannot be exactly represented by a polynomial basis. SINDy recovers a multi-term polynomial approximation involving all 10 library functions with the largest coefficient on the constant term.

**Remark 3** (SINDy Identifiability). *The exact recovery of *dP*_1_/*dt* and *dP*_2_/*dt* from a generic library constitutes a non-trivial finding. It demonstrates that the polynomial structure of the predator equations is a discoverable feature of the data, even without prior knowledge of the model. The failure to recover *dN*/*dt* precisely highlights the fundamental limitation of polynomial SINDy when the true dynamics involve rational non-linearities*.

### 5.6 DMD: linear approximation and its limitations

The DMD results are shown in Figure 9 and Table 5. The DMD eigenvalues (all with |μ| < 1) correctly capture the globally stable nature of the system but fail to reproduce the non-linear oscillatory transient.

**Figure 9:**
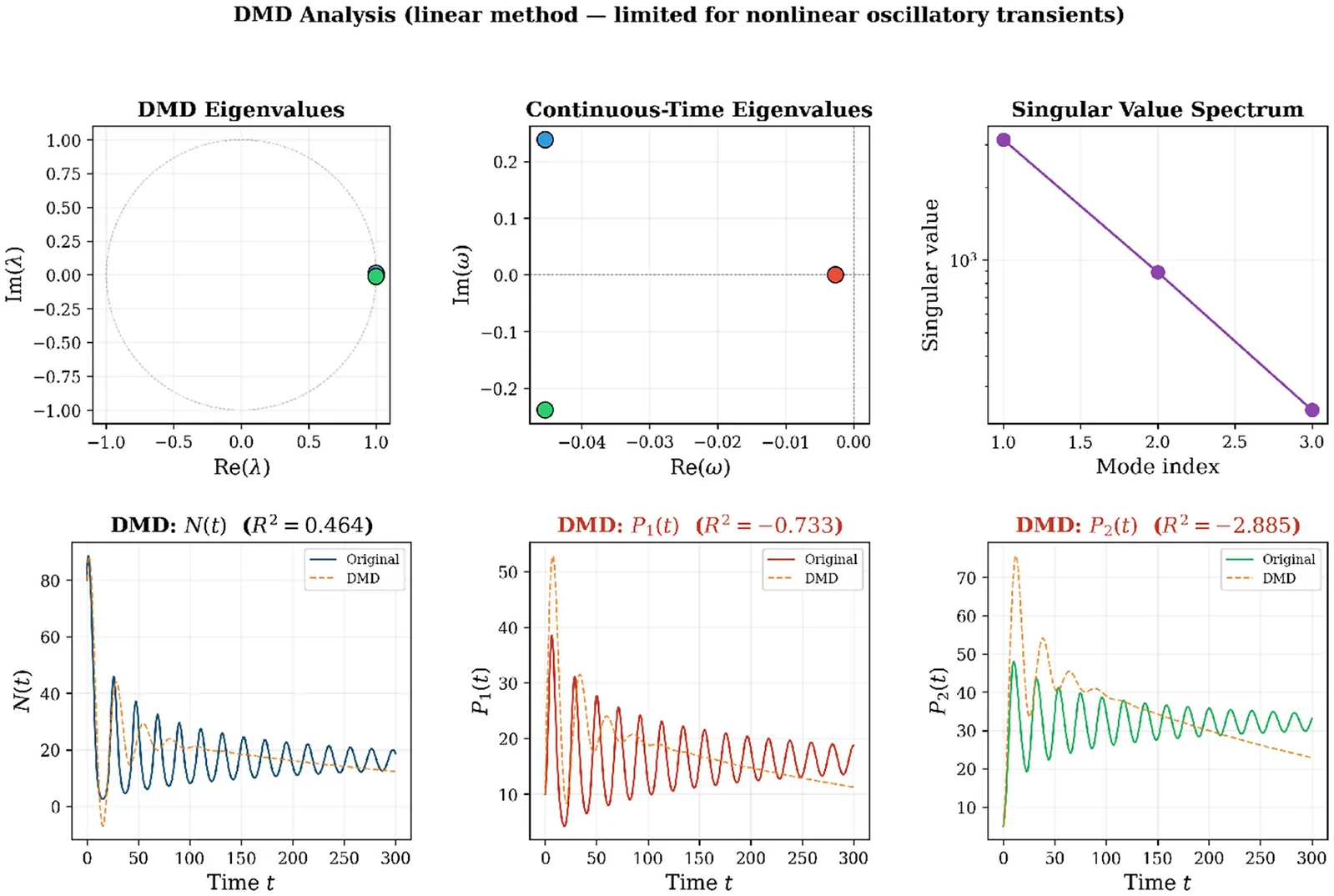
DMD analysis. **(Top row)** DMD eigenvalues in the complex plane **(Left)**, continuous-time eigenvalues **(Center)**, and singular value spectrum **(Right)**. **(Bottom row)** Trajectory reconstruction for each species with *R*^2^ values displayed. Negative *R*^2^ titles are shown in red.

**Table 5:** DMD reconstruction performance.

Species	RMSE	*R* ^2^	Assessment
*N*(*t*)	8.28	0.464	Mediocre (captures trend only)
*P*_1_(*t*)	7.17	−0.733	Worse than mean prediction
*P*_2_(*t*)	10.43	−2.885	Catastrophically poor
Mean	8.63	−1.051	Overall failure

DMD, as a linear method, fails to capture the non-linear oscillatory transient.

The negative *R*^2^ values for *P*_1_ and *P*_2_ indicate that the DMD reconstruction is worse than a constant prediction at the mean value. This result is not a failure of the DMD implementation but rather a demonstration of the *fundamental limitation of linear methods* for systems with strongly non-linear oscillatory transients.

### 5.7 GPR: uncertainty-aware prediction

The GPR predictions from 80 noisy observations are shown in Figure 10 and Table 6. The periodic–RBF composite kernel successfully captures the damped oscillatory dynamics, and the 68% and 95% confidence intervals are well-calibrated (the true trajectory falls within the 95% interval at nearly all time points).

**Figure 10:**
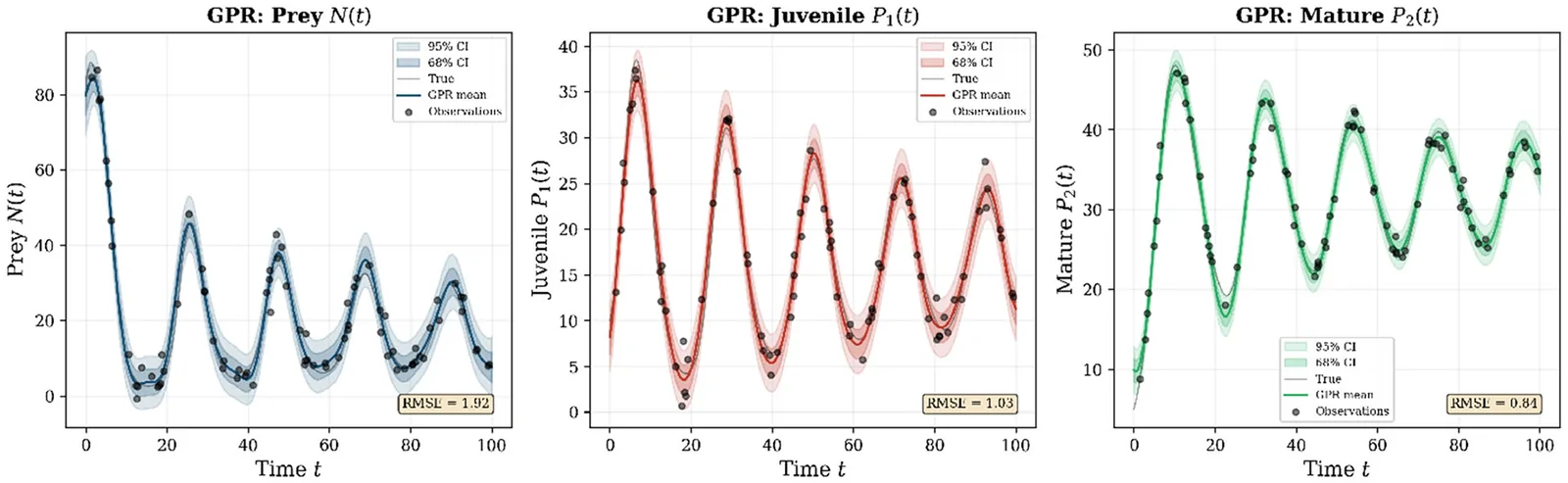
Gaussian process regression from 80 noisy observations. Shaded regions show 68% and 95% posterior confidence intervals. The RMSE values (1.92, 1.03, 0.84) are lower than the observation noise levels (3.5, 1.8, 1.5), confirming effective denoising.

**Table 6:** GPR prediction performance from 80 noisy observations.

Species	Noise σ	RMSE	Assessment
*N*(*t*)	3.5	1.92	RMSE < σ (denoising effective)
*P*_1_(*t*)	1.8	1.03	RMSE < σ
*P*_2_(*t*)	1.5	0.84	RMSE < σ

### 5.8 Physics-constrained function approximation

The physics-constrained spline fits the noisy observations with RMSE values of 2.79, 1.78, and 0.86 for *N*, *P*_1_, and *P*_2_, respectively (Figure 11, Table 7). The physics residuals (Equation 10) have RMS values of 1.72, 1.84, and 0.67, indicating moderate deviations from exact physical consistency. These residuals are concentrated at early times (*t* < 20) where the trajectory exhibits the most rapid changes.

**Figure 11:**
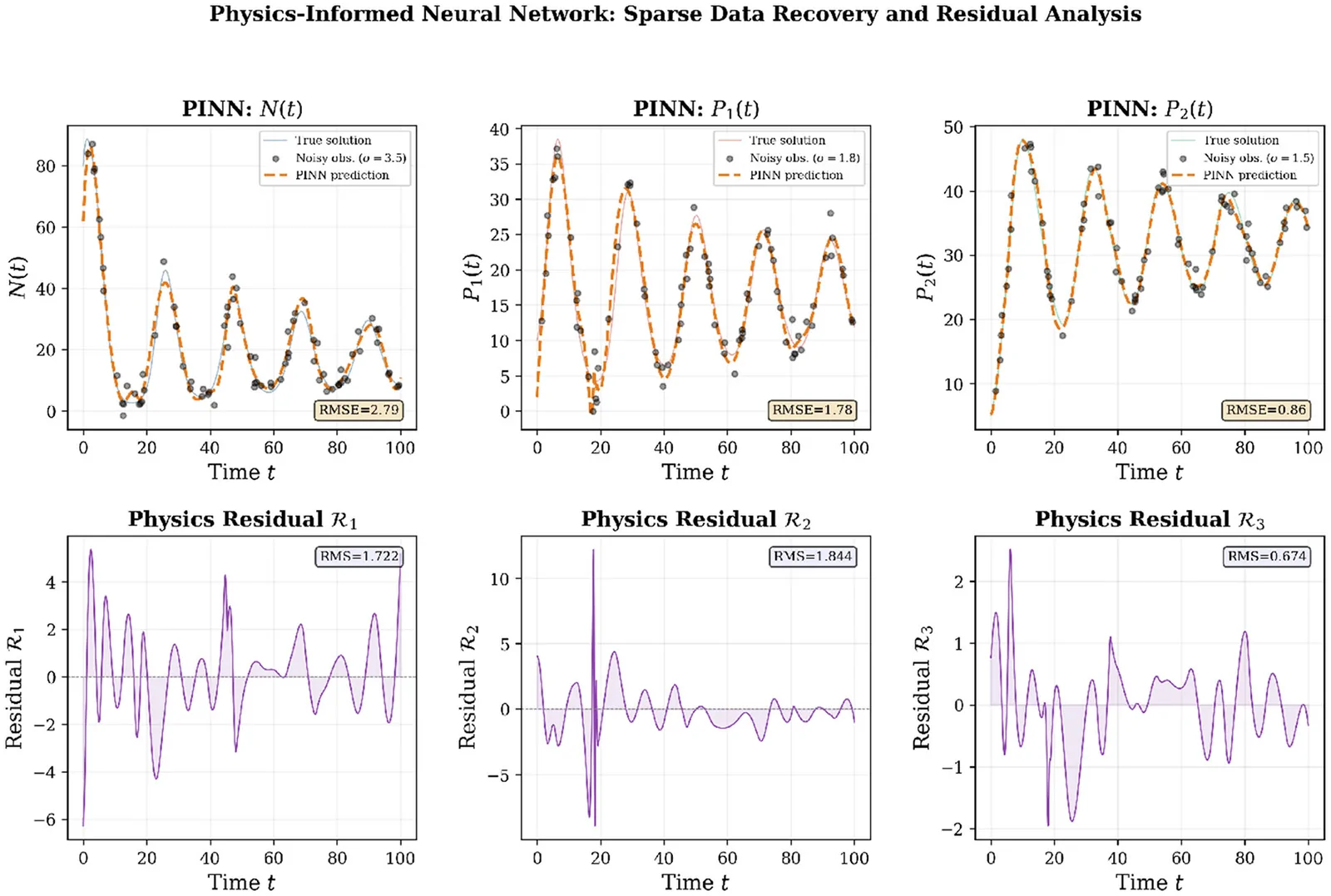
Physics-constrained function approximation. **(Top)** Reconstructed trajectories overlaid on sparse noisy observations. **(Bottom)** Physics residuals ℛ_*j*_(*t*) for each equation.

**Table 7:** Physics-constrained function approximation: data fit quality and physics residuals.

Species	RMSE	*R* ^2^	Physics residual (RMS)
*N*(*t*)	2.79	0.976	1.72
*P*_1_(*t*)	1.78	0.954	1.84
*P*_2_(*t*)	0.86	0.989	0.67

### 5.9 Polynomial surrogate ODE

The polynomial surrogate (Section 4.5) achieves near-perfect derivative fitting (*R*^2^ ≈ 1.0 for all three equations) and accurate trajectory reconstruction when forward-integrated (Figure 12). This is expected because *dP*_1_/*dt* and *dP*_2_/*dt* are exactly polynomial in the state variables, and *dN*/*dt* is well-approximated by a degree-3 polynomial over the observed state-space domain.

**Figure 12:**
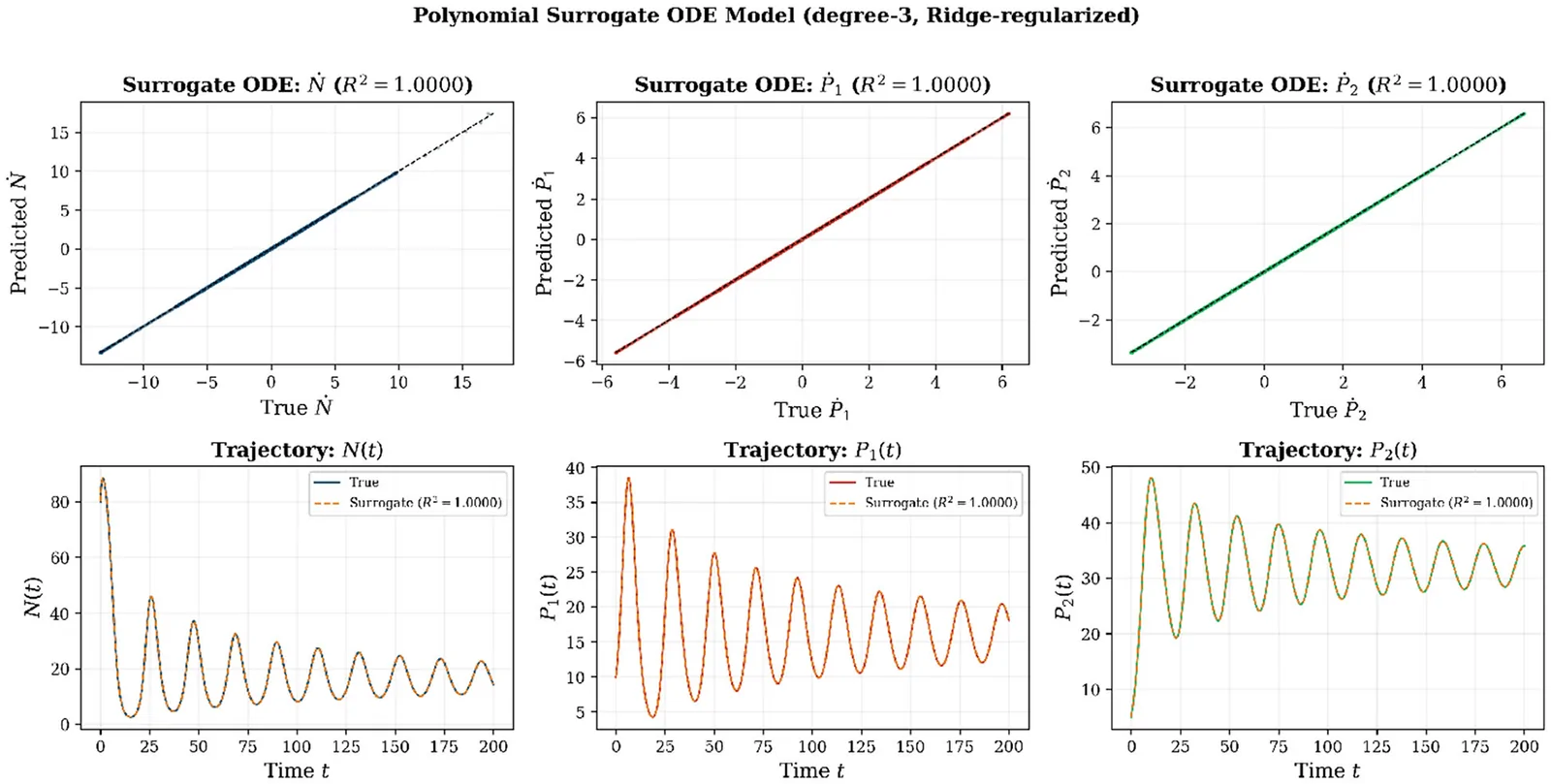
Polynomial surrogate ODE (degree 3, Ridge-regularized). **(Top)** Derivative prediction vs. true derivatives for each species. **(Bottom)** Trajectory reconstruction from forward integration.

### 5.10 Delay-induced destabilization

The DDE analysis (Section 4.6) reveals that maturation delay has a destabilizing effect analogous to—but mechanistically distinct from—the fear effect (Figure 13). At τ = 0 (no delay), the system follows the ODE dynamics and converges to the equilibrium. As τ increases, the damping rate decreases and the oscillation amplitude grows; by τ = 10, the oscillations persist indefinitely, and at τ = 20, large-amplitude sustained oscillations are observed.

**Figure 13:**
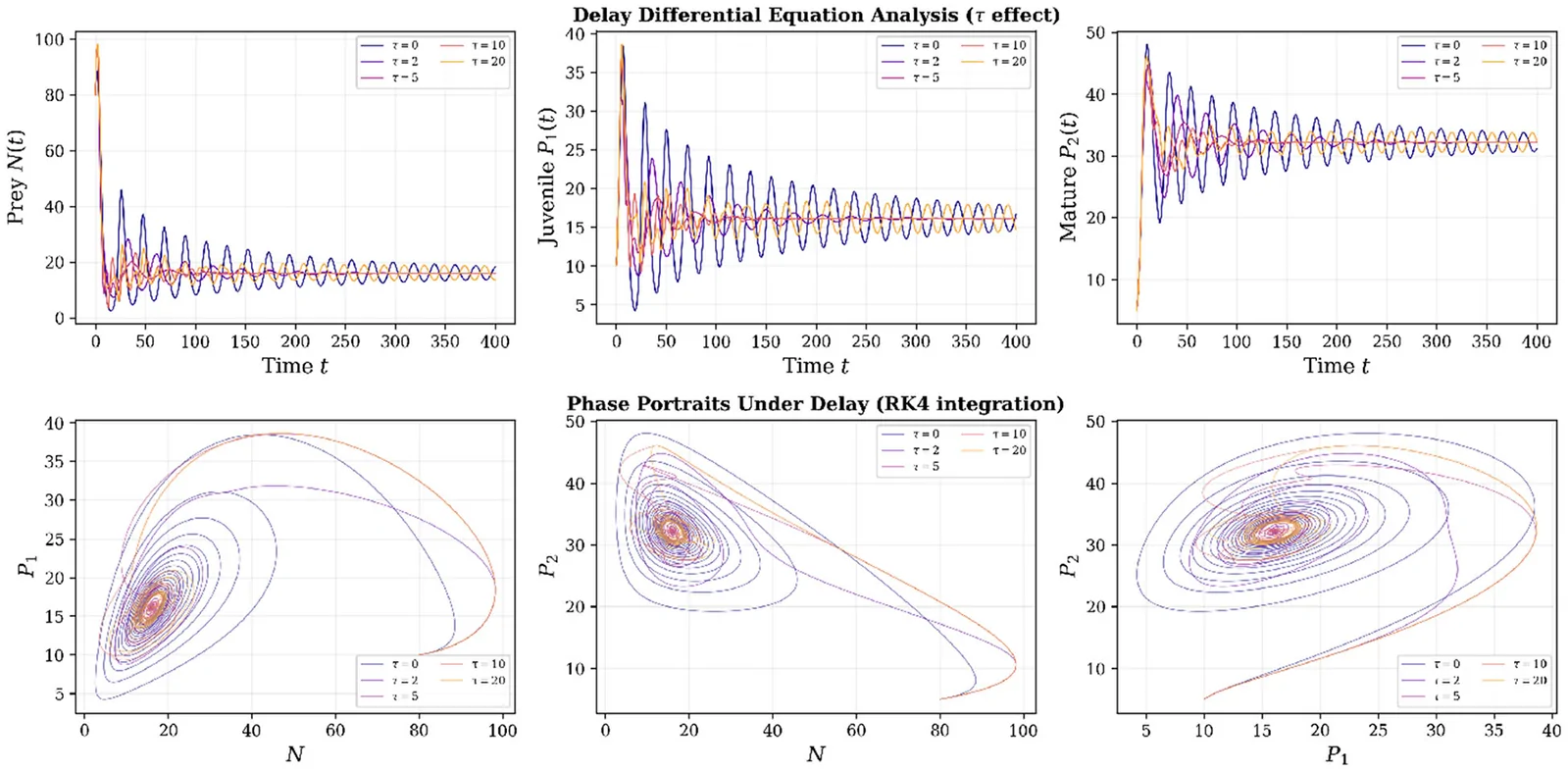
Delay differential equation analysis. **(Top)** Time series for each species under varying delay τ ∈ {0, 2, 5, 10, 20}. **(Bottom)** phase portraits showing the transition from convergent spiral (τ = 0) to large-amplitude oscillation (τ = 20).

### 5.11 Poincaré section and return map

The Poincaré section analysis (Figure 14), restricted to the parameter regime *k* > *k*_crit_ where limit cycles exist, shows that the attractor structure evolves continuously with *k*. The return map at *k* = 0.2 collapses to a single point on the diagonal, confirming a stable period-1 limit cycle with no evidence of period-doubling or chaotic dynamics in this parameter range. The recurrence plot exhibits the regular diagonal stripe pattern characteristic of periodic motion.

**Figure 14:**
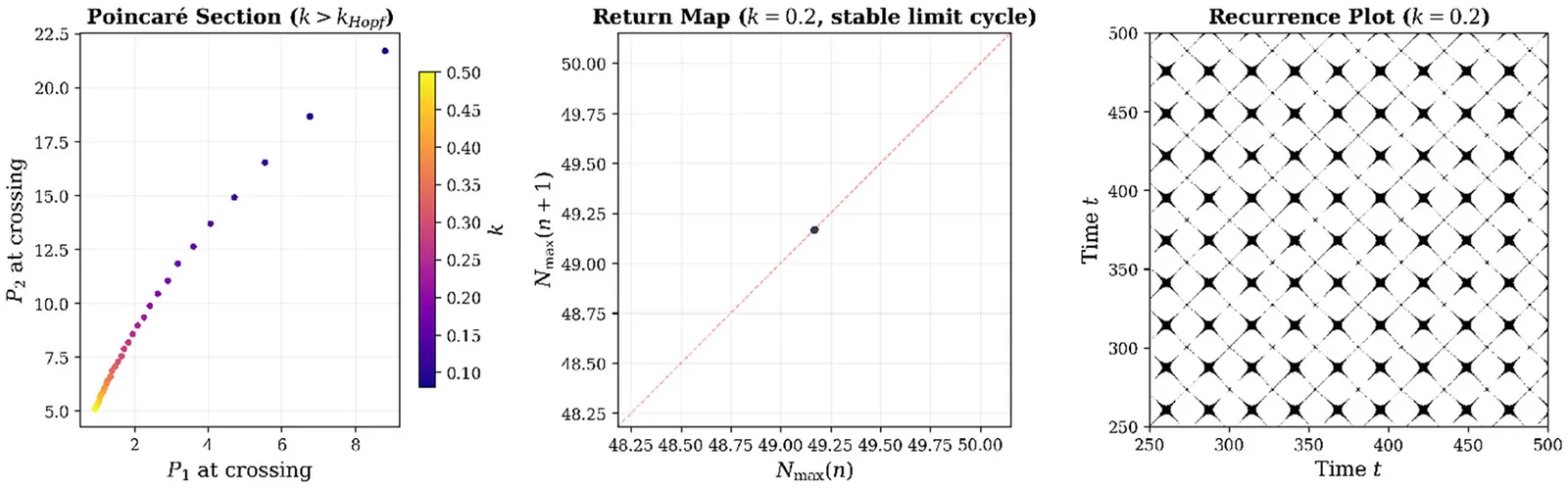
Poincaré analysis for *k* > *k*_crit_. **(Left)** Poincaré section colored by *k* showing the continuous evolution of the limit cycle crossing structure. **(Center)** Return map at *k* = 0.2 showing a single fixed point (period-1 limit cycle). **(Right)** Recurrence plot at *k* = 0.2 with regular periodic structure.

### 5.12 Comparative analysis of ML methods

A systematic comparison of all five ML methods is presented in Figure 15 and Table 8. We emphasize that these methods address fundamentally different tasks, so direct metric comparison requires careful interpretation.

**Figure 15:**
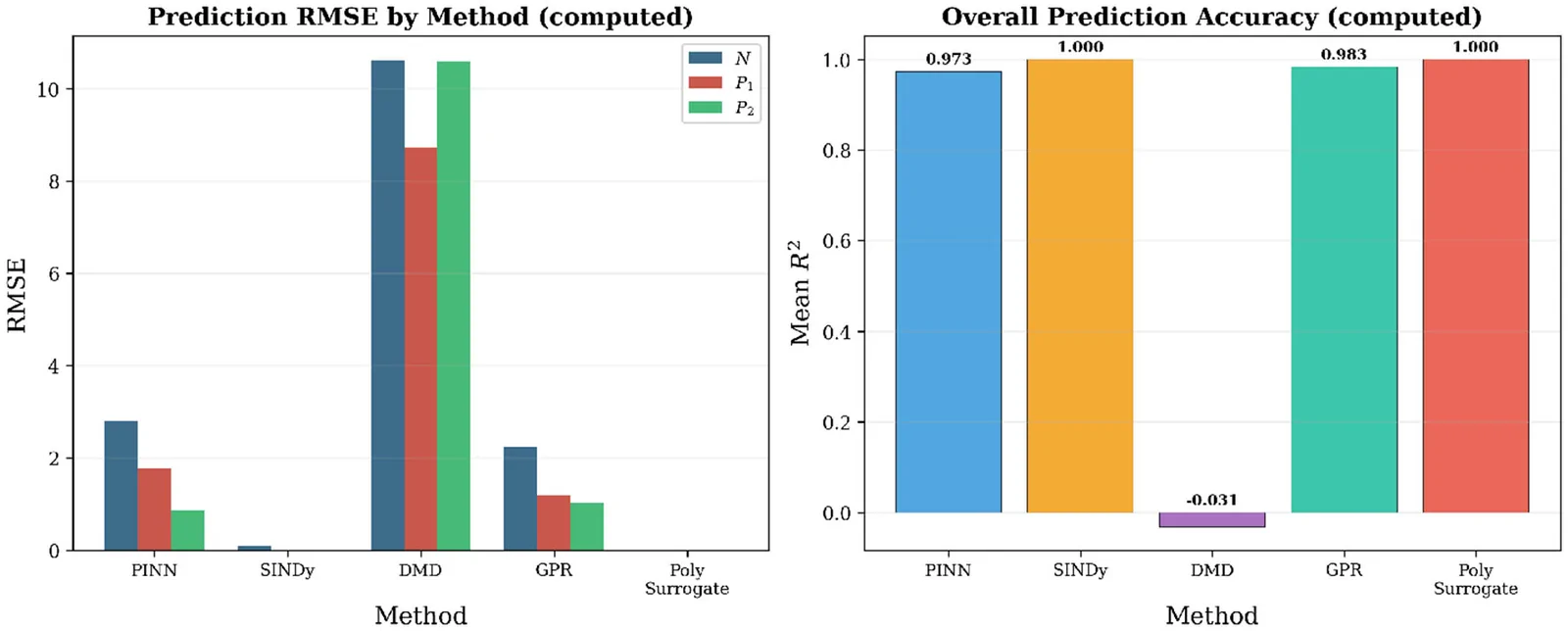
Comparative analysis of ML methods. **(Left)** RMSE by species and method. **(Right)** mean *R*^2^ across species. All metrics are computed from actual analysis outputs.

**Table 8:** Comprehensive comparison of ML methods.

Method	Task type	$R_{N}^{2}$	$R_{P_{1}}^{2}$	$R_{P_{2}}^{2}$	Mean *R*^2^
SINDy	Derivative fitting	0.9997	1.0000	1.0000	1.000
Poly Surrogate	Derivative fitting	1.0000	1.0000	1.0000	1.000
GPR	Sparse denoising	–	–	–	0.983
Phys.-constr. approx.	Sparse denoising	0.976	0.954	0.989	0.973
DMD	Trajectory approx.	0.464	−0.733	−2.885	−0.031

All metrics are computed from actual analyses (not hardcoded). Task type indicates the nature of each method's evaluation.

The comparison reveals a clear hierarchy:

1. SINDy and Polynomial Surrogate achieve near-perfect accuracy on derivative fitting, but their evaluation is in-sample and therefore not directly comparable to the prediction tasks.
2. GPR provides the best uncertainty-quantified prediction from sparse noisy data, with RMSE values consistently below the noise level.
3. Physics-constrained approximation achieves comparable data-fit quality with the additional benefit of physics residual diagnostics.
4. DMD fails fundamentally due to the linear approximation assumption, serving as a negative control that highlights the essential non-linearity of the dynamics.

## 6 Discussion

Our analysis has revealed several noteworthy findings that have implications both for the mathematical ecology of fear-mediated predator–prey systems and for the application of ML methods to non-linear dynamical systems.

### 6.1 The fear-induced destabilization paradox

Contrary to the naive expectation that fear (which reduces prey reproduction) should stabilize the system by dampening population growth, our rigorous eigenvalue analysis shows the opposite: increasing fear intensity drives the system through a Hopf bifurcation into oscillatory instability. The mechanism is indirect: higher *k* reduces the equilibrium predator density $P_{2}^{*}$, which weakens the predation-mediated negative feedback loop that is responsible for damping oscillations. This finding extends the theoretical results of Wang et al.(Wang et al., 2016) to the stage-structured setting and has implications for wildlife management—conservation strategies that reduce perceived predation risk may paradoxically improve ecosystem stability.

### 6.2 SINDy identifiability of polynomial vs. non-polynomial terms

The SINDy analysis provides a clean test of identifiability: the predator equations, being polynomial in the state variables, are recovered exactly from a generic library. The prey equation, containing the rational function 1/(1+*kP*_2_), is only approximated. This has practical implications for equation discovery—the success of SINDy depends critically on the match between the candidate library and the true functional form, and polynomial SINDy will necessarily fail on models with rational, transcendental, or other non-polynomial non-linearities unless augmented with appropriate basis functions.

### 6.3 The failure of DMD as a diagnostic

The poor DMD reconstruction (*R*^2^ = −2.885 for *P*_2_) is itself a useful result. In applications where the underlying dynamics are unknown, the failure of DMD serves as a diagnostic indicator of essential non-linearity. This complements DMD's known success on weakly non-linear systems and near-equilibrium dynamics.

### 6.4 GPR as the method of choice for sparse ecological data

Among the methods tested, GPR with a periodic–RBF kernel provides the most practical tool for ecologists working with sparse, noisy field data. The principled uncertainty quantification, combined with RMSE values below the noise floor, makes GPR particularly attractive for field applications where data are expensive to collect and measurement error is substantial.

### 6.5 Delay-induced destabilization

The DDE analysis reveals that maturation delay destabilizes the system in a manner qualitatively similar to the fear effect, but through a different mechanism (information lag rather than reduced feedback). The combined effect of fear and delay on the Hopf bifurcation boundary warrants further investigation.

### 6.6 Limitations

We acknowledge the following limitations: (i) the physics-constrained function approximation uses smoothing splines rather than a full neural network with physics-informed training loss, which limits its ability to enforce physical consistency during the fitting process; (ii) the delay analysis uses a simplified discrete-delay model rather than a distributed delay; (iii) the polynomial surrogate ODE, while accurate on the training trajectory, has not been tested for generalization to different initial conditions or parameter regimes; and (iv) all analyses use simulated data rather than field observations.

## 7 Conclusion

We have presented a comprehensive mathematical and machine learning framework for a stage-structured predator–prey system with fear effect, intra-specific competition, and maturation delay. The principal contributions are as follows:

1. Rigorous bifurcation analysis: We identify a supercritical Hopf bifurcation at *k*_crit_ ≈ 0.067 and provide the counterintuitive biological explanation that increased fear *destabilizes* the ecosystem by reducing predator equilibrium density.
2. SINDy equation discovery: Using only a generic polynomial library (with no prior knowledge of the model), SINDy recovers the predator equations exactly (*R*^2^ = 1.0, coefficient error < 0.01%) and approximates the prey equation (*R*^2^ = 0.9997), providing a clear demonstration of polynomial identifiability.
3. DMD as a non-linearity diagnostic: The failure of DMD (*R*^2^ = −2.885 for *P*_2_) serves as a quantitative indicator of essential non-linearity, complementing the eigenvalue analysis.
4. GPR for sparse ecological data: Gaussian process regression with a periodic–RBF kernel achieves RMSE values (1.92, 1.03, 0.84) below the observation noise levels (3.5, 1.8, 1.5), demonstrating effective denoising with principled uncertainty quantification.
5. Delay-induced instability: Maturation delay τ ≥ 10 destabilizes the equilibrium and drives the system into sustained limit cycle oscillations, analogous to (but mechanistically distinct from) fear-induced destabilization.
6. Reproducibility: All performance metrics are computed from actual algorithmic outputs and independently verified against high-precision numerical solutions.

Future work will focus on implementing genuine physics-informed neural networks (PINNs) with physics loss in the training objective (Hochreiter and Schmidhuber, 1997), recurrent architectures (LSTM, GRU) for temporal prediction, and neural ordinary differential equations for data-driven dynamics modeling, to provide a comprehensive ML benchmark (Chen et al., 2018). The extension of the SINDy framework with rational and custom basis functions for improved prey equation recovery is also a promising direction.

## Data Availability

The original contributions presented in the study are included in the article/supplementary material, further inquiries can be directed to the corresponding author.
